# The prognostic value of clonal heterogeneity and quantitative assessment of plasma circulating clonal IG-VDJ sequences at diagnosis in patients with follicular lymphoma

**DOI:** 10.18632/oncotarget.14448

**Published:** 2017-01-02

**Authors:** Clémentine Sarkozy, Sarah Huet, Victoria E.H Carlton, Bettina Fabiani, Alain Delmer, Fabrice Jardin, Marie-Helene Delfau-Larue, Maya Hacini, Vincent Ribrag, Stéphanie Guidez, Malek Faham, Gilles Salles

**Affiliations:** ^1^ Hospices Civils de Lyon, Centre Hospitalier Lyon-Sud, Service d’Hématologie, 69495 Pierre Bénite Cedex, France; ^2^ NSERM1052, CNRS 5286, Université Claude Bernard, Faculté de Médecine Lyon-Sud Charles Mérieux Lyon-1, 69495 Pierre Bénite Cedex, France; ^3^ Hospices Civils De Lyon, Laboratoire d’Hématologie, Pierre Bénite, France; ^4^ Adaptive Biotechnologies Corp., South San Francisco, CA, USA; ^5^ Assistance Publique – Hôpitaux de Paris, Hôpital Saint-Antoine, Paris, France; ^6^ Service d’Hématologie, CHU de Reims, Reims, France; ^7^ Centre Henri Becquerel, Service d’Hématologie, Rouen, France; ^8^ Department of Biological Hematology and Immunology, Assistance Publique – Hôpitaux de Paris, Groupe Hospitalier Mondor, Créteil, France; ^9^ Centre Hospitalier de Chambery, Service d’Hématologie, Chambery, France; ^10^ Institut Gustave Roussy, Service d’Hématologie, Université Paris-Sacley, Villejuif, France; ^11^ Service d’Hématologie, CHU de Poitiers, France

**Keywords:** follicular lymphoma, circulating tumor DNA, prognostic factor, rituximab, maintenance

## Abstract

Recent advances in next-generation sequencing (NGS) have enabled the quantitation of circulating tumour DNA (ctDNA) encoding the clonal rearranged V(D)J immunoglobulin locus. We aimed to evaluate the clonal heterogeneity of follicular lymphoma (FL) in the tumour and the plasma at diagnosis and to assess the prognostic value of the ctDNA level. Plasma samples at diagnosis were available for 34 patients registered in the PRIMA trial (NCT00140582). One tumour clonotype or more could be detected for 29 (85%) and 25 (74%) patients, respectively, in the tumour or plasma samples. In 18 patients, several subclones were detected in the tumour (2 to 71 subclones/cases) and/or in the plasma (2 to 20 subclones/cases). In more than half of the cases, the distribution of subclones differed between the tumour and plasma samples, reflecting high clonal heterogeneity and diversity in lymphoma subclone dissemination. In multivariate analysis, a high level of ctDNA was the only independent factor associated with patients’ progression-free survival (HR 4, IC 95 (1.1-37), p=.039). In conclusion, an NGS-based immunosequencing method reveals the marked clonal heterogeneity of follicular lymphoma in patients with FL, and quantification of ctDNA at diagnosis represents a potential powerful prognostic biomarker that needs to be investigated in larger cohorts.

## INTRODUCTION

Follicular lymphoma (FL) is a clinically heterogeneous disease, with some patients presenting with a high tumour burden, usually with highly disseminated disease at diagnosis, and others a low tumour burden without significant symptoms for several years [1]. Response to treatment is also heterogeneous, and while some patients will remain disease-free after a combination of anti-CD20 monoclonal antibody and cytotoxic agents, eventually prolonged with anti-CD20-based maintenance therapy, others experience early disease progression and may develop chemo-refractoriness. This clinical heterogeneity certainly depends on the biological heterogeneity of the disease. Although FL tumour cells share identical immunoglobulin (*IG*) gene rearrangements, they also present different patterns of somatic mutations in the *IG* gene sequence, revealing extensive clonal heterogeneity [2]. Whether this Ig clonal heterogeneity is also a pattern of the oncogenic genetic events has been recently investigated, thanks to fluorescence activated cell sorting and next-generation sequencing (NGS) technologies [3]. Using immunoglobulin somatic mutations and by comparing diagnosis and relapse pairs, clonal evolution could be determined. The pattern of associated oncogenic genetic events revealed a high intra-clonal heterogeneity and allowed the distinction of early and late genetic events during disease evolution and lymphomagenesis. The framework exposed in recent studies on clonal diversity clearly provides insight into lymphoma development and might potentially account for FL progression and/or transformation [3–5]. If information on the mutational pattern in FL is not routinely available, clonal diversity might represent a future biomarker of interest if this heterogeneity is related to clinical features.

In solid tumor, intratumoral diversity analysis has become accessible by using circulating tumor DNA (ctDNA) as primary material [6]. Indeed, apoptosis and necrosis of the malignant tumour cells provoke the release of tumour DNA into blood circulation, which can be detected and quantified by NGS in order to assess the tumour dynamics. Recent advances in NGS techniques enable the quantitation of circulating tumour DNA (ctDNA) encoding the clonal rearranged V(D)J Ig receptor gene sequence of tumour cells [7]. The unique malignant V(D)J Ig gene sequence is then called the tumour clonotype. This non-invasive technique, developed by Adaptive Biotechnologies® technology, allows the quantification of the circulating tumour clonotype in diffuse large B cell lymphoma (DLBCL), mantle cell lymphoma (MCL), chronic lymphocytic leukaemia (CLL), multiple myeloma and acute lymphoid leukaemia and can be used for minimal residual disease (MRD) detection [7–14]. Proof of concept has even been reported in classical Hodgkin lymphoma [15]. More recently, this approach was used to reveal the importance of oligoclonality and ongoing hypermutation in CLL-like monoclonal B-cell lymphocytosis (MBL) [16].

The aims of this study were first to evaluate the clonal heterogeneity of FL using an NGS-based immunosequencing method and to evaluate its distribution in tumour and plasma samples at diagnosis in patients included in the PRIMA trial [17]. We then assessed the prognostic value of the level of ctDNA and the clonal heterogeneity at diagnosis for progression-free survival (PFS).

## RESULTS

### Evidence for ongoing somatic hyper-mutation (SHM) and tumour dissemination by clonotype detection

**Figure 1:**
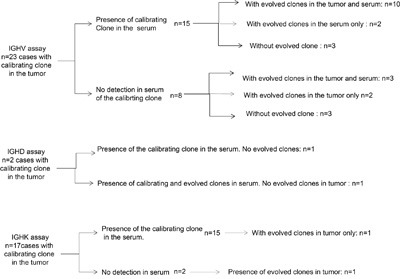
Identification of the calibrating clone and repartition of the different subclones in the tumour and in the plasma with the IGH-V, IGH-D and IGK assays

One tumour clonotype could be detected in 29 patients (85%) in the diagnostic tumour sample (*IGH-V*, n=23; *IGH-D*, n=2; *IG-K*, n=17). Clonotype ctDNA could also be detected in 25/29 (86%) match plasma samples (Figure 1). The level of clonotype ranged from 0 to 388,821 clonotypes per million diploid genomes in these 29 plasma samples (median 39,720 clonotypes per million diploid genome). The initial clinical and biological characteristics of these patients and of all the PRIMA patients are described in Table 1.

**Table 1:** Initial clinical and biological characteristics of the 34 patients included in the analysis and of the 29 patients with a clonotype identified in the tumour biopsy at diagnosis

Patients’ Characteristics	PRIMA1135 patients	Plasma + Tumour DNA34 patients	Clonotype identified29 patients
**Gender (female)**	545 (48%)	13 (38%)	10 (34%)
**Age (median, min, max) >60 y**	57 years (23 – 85)402 (35%)	55 years (28-74)11 (32%)	55 years (28-74)9 (31%)
**B symptoms**	363 (32%)	11/34 (32%)	10/29 (34%)
**Bulky disease**	518/1112 (47%)	15/34 (41%)	13/29 (45%)
**FLIPI score**			
**0-1**	239 (21%)	6 (18%)	5 (18%)
**2**	405 (36%)	14 (41%)	12 (41%)
**3-5**	489 (43%)	14 (41%)	12 (41%)
**Haemoglobin <12 g/dL**	227 (20%)	9/34 (26%)	8/29 (28%)
**LDH > UNV**	378 (34%)	8/33 (24%)	6/28 (21%)
**β2 microglobulin >=3**	341 (33%)	14/34 (41%)	13/29 (45%)
**Ann Arbor Stage**			
**I-II**	109 (10%)	1 (3%)	0 (0%)
**III-IV**	1026 (90%)	33 (99%)	29 (100%)
**Bone marrow involvement**	635/1101 (56%)	20/31 (66%)	19/27 (70%)
**ECOG-PS >=1**	413 (36%)	14 (41%)	12 (41%)
**Presence of circulating lymphoma cells**	92/1013 (9%)	5/32 (16%)	4/27 (15%)

Bulky disease is defined as a nodal or extra-nodal (except spleen) mass > 7 cm in its greater diameter.

Among the 24 patients with calibrating clonotype(s) detected by one of the *IGH* assays (-V or –D, one patient being positive with both assays), the presence of evolved clones was detected in tumour or plasma samples in 18 cases and in both in 13 cases, revealing substantial sub-clonal dissemination (2 to 71 different subclones per patient in tumours and 2 to 20 in plasma). In 6 cases, no evolved clonotype was detected in the tumour sample, but ctDNA was found in all 6 patients with evolved clonotypes in 3 out of these 6 cases. In 2 cases, evolved clones were detected in the tumour, but no ctDNA was detected in the plasma. Finally, 3 patients had a unique clonotype without subclones in the tumour biopsy and without detectable ctDNA. In these 3 latter cases, the number of lymphoma clonotype molecules detected in the tumour was high (587,100; 919,400 and 164,400 of lymphoma clonotype molecules per million diploid genomes, respectively), making it unlikely that a lack of sensitivity hampered the detection of evolved clones. Altogether, the presence of ctDNA originating from at least 2 subclones was revealed with *IGH* assays in 16/24 cases (67%). The *IGK* assay allowed the detection of clonotypes in 17 patients. Most of the patients (14/17) had the same clonotype in the plasma, without evolved clones. Only one of these patients presented evolved clones both in the tumour and in the plasma, with 2 different subclones in the tumour and 7 in the plasma.

### Distribution of the different clonotypes between the tumour and plasma

**Figure 2:**
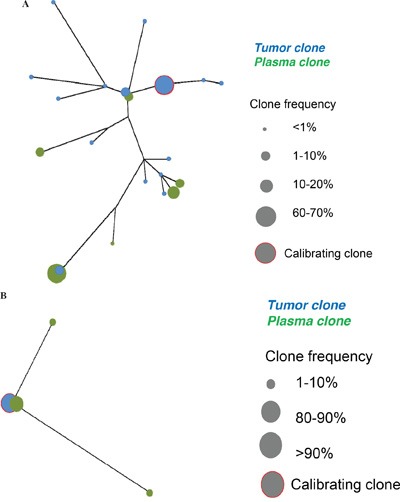
**A** and **B.** Two examples illustrating the clonal heterogeneity of FL and the heterogeneous distribution of clonal and subclonal populations between the tumour biopsy and the plasma.

Among the 19 cases with circulating clonotypes detected by the *IGH* (*-V*, n=18 and/or *-D*, n=2) assays, the distribution of the different clonotypes differed in some cases between the tumour and plasma samples. Indeed, in 11 cases, an evolved clone different from the tumour calibrating clone was the highest-frequency clone in the plasma (Figure 2A). Similarly, in 3 cases, the presence of evolved clonotypes was detected in the plasma, whereas the calibrating clonotype was the only clone found in the tumour (Figure 2B). These results suggest that the different subclones present a striking heterogeneity in their capacity to disseminate outside the tumour niche. We then investigated whether the disseminating clones harboured a specific *IGH* sequence that might be responsible for stronger interactions with the microenvironment, as previously suggested [18]. We could not find a correlation between the presence of detectable ctDNA and a stereotyped *IGH* sequence or a particular N-glycosylation motif within the CDR3 region of the *IGH* clonotypes (not shown).

### Clinical correlations and prognostic value of the presence of ctDNA (Table 2)

The level of clonotype ctDNA ranged from 0 to 388,821 clonotypes per million diploid genomes in the 29 plasma samples from patients with a calibrating clonotype detected in the tumours. As shown in Table 2, the presence or absence of ctDNA was associated with bone marrow involvement (19/19 patients with bone marrow involvement had detectable ctDNA versus 5/8 patients without bone marrow involvement, p=.005, χ^2^ test), but not with the FLIPI score, the serum LDH level, anaemia, bulky disease, the presence of circulating lymphoma cells (detected by morphology or flow examination) or the β2 microglobulin level. The level of the highest-frequency clone of ctDNA in the plasma diagnostic sample of each patient (higher or lower than the median value for each assay) were also significantly associated with the presence of circulating lymphoma cells (circulating lymphoma cells were present in 4/14 patients with ctDNA level higher than the median value versus 0/14 for patients with ctDNA level lower than the median value, p=.024, χ^2^ test) and LDH value (p=.04), but not with the other clinical characteristics.

**Table 2:** Statistical correlations between the initial clinical and biological characteristics and the presence or absence of ctDNA and the level of ctDNA (higher or lower than the median value)

Characteristics (N=29 patients)	Presence of ctDNA (N=25/29)	ctDNA > median (N=14/29)
**FLIPI score:3-5 (n=12) versus 1-2 (n=17)**	11/12 versus 14/17, p=.47	7/12 versus 7/17, p=.36
**Circulating lymphoma cells: present (n=4) versus absent (n=23)**	4/4 versus 20/23, p=.44	4/4 versus 9/23, p=.024
**Bone marrow:involved (n=19) versus not involved (n=8)**	19/19 versus 5/8, p=.005	12/19 versus 2/8, p=.07
**LDH:>UNV (n=6) versus ≤ UNV (n=22)**	5/6 versus 19/22, p=.85	5/6 versus 8/22, p=.04
**Bulky disease:yes (n=13) versus no (n=16)**	11/13 versus 14/16, p= .82	5/13 versus 9/16, p=.34
**Anaemia:Haemoglobin <12 g/dL (n=8) versus ≥ 12 g/dL (n=21)**	7/8 versus 18/21, p=.9	6/8 versus 8/21, p=.08
**β2 microglobulin:≥ 3 mg/L (n=13) versus <3 mg/L (n=16)**	12/13 versus 13/16, p=.39	8/13 versus 6/16, p=.19

**Figure 3:**
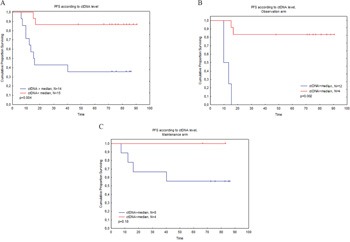
**A.** PFS according to the level of ctDNA in 29 patients with detectable calibrating clone presence in the tumour. **B.** PFS according to the level of ctDNA in the PRIMA observation subgroup. **C.** PFS according to the level of ctDNA in the PRIMA rituximab-maintenance subgroup.

Next, we assessed the prognostic value of the level of ctDNA, using as the threshold the median ctDNA level for each assay. The 14 patients with higher levels of ctDNA at diagnosis experienced a significantly shorter PFS than the 15 patients with lower levels of ctDNA (median 15.3 months vs. not reached, p=.004, Figure 3A). In univariate prognostic analysis, bone marrow involvement and the presence of circulating lymphoma cells had no significant impact on PFS (p=0.33 and 0.54 respectively). As ctDNA value is correlated with bone marrow involvement and with the presence of circulating lymphoma cells, we performed an exploratory multivariate Cox regression model including FLIPI score, bone marrow involvement, presence of circulating lymphoma cells and ctDNA level. High ctDNA level was the only factor significantly associated with a worse PFS (HR 6.2, IC 95 (2-162), p=.001) (p=0.14, 0.52 and 0.25 for FLIPI, bone marrow involvement and presence of circulating lymphoma cells respectively). When including LDH value in the model, ctDNA level remained the only factor associated with outcome (data not shown).

We next further analyzed the prognostic impact of a high level of ctDNA according to the randomization arm (rituximab maintenance or observation). In the observation arm (Figure 3B), the 4 patients with a high ctDNA level had a median PFS of only 9.8 months versus not reached for the 12 patients with a low ctDNA level (p=.002). In contrast, in the maintenance arm (Figure 3C), the adverse prognostic value of elevated ctDNA levels was apparently erased, with no PFS difference among the 8 patients with a high ctDNA value and the 4 patients with a low ctDNA value (p=0.18).

### Clonal heterogeneity and patient outcome

**Figure 4:**
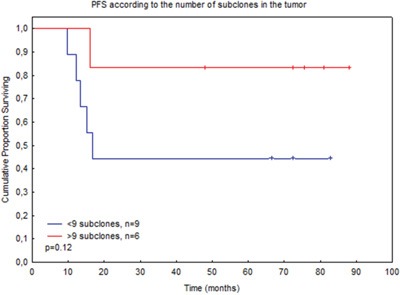
PFS according to the number of subclones identified in the tumour biopsy

We then looked at the impact of clonal heterogeneity on the patients’ outcomes. Among the 23 patients with an IGH-V clonotype in the tumour sample, 15 presented evolved subclones, ranging from 2 to 71 subclones, with a median of 9 subclones per patient biopsy. Among the 12 patients with an IG-K clonotype, only two presented one subclone, and these two also had an IGH-V clonotype (with 14 and 4 subclones, respectively). The patient presenting an IGH-D without an IGH-V clonotype had no subclones detected in the tumour sample. Therefore, among the 29 patients with a tumour clonotype detected in the biopsy sample, 15 presented at least one subclone in the tumour sample, with a median of 9 subclones, and 14 had no subclones. Among these 15 patients, a non-significant trend towards a shorter PFS for patients with a lower number of subclones was observed. Indeed, the 6 patients presenting more than 9 subclones had a 6-year PFS of 83% (SE 15%), compared to 44% (SE 16%) for the 9 patients presenting less than 9 subclones (p=.12, Figure 4). For the plasma samples, 15 patients with an IGH-V clonotype presented subclones, 1 with an IGH-D clonotype and none with an IG-K clonotype. In these 16 patients, the number of subclones detected in the plasma varied from 2 to 20 (median 2). There was no difference in PFS between the 7 patients presenting more than 2 subclones and the 9 presenting less than 2 subclones (p=.85).

## DISCUSSION

We report here the first series using next-generation sequencing of rearranged *IGH* and *IGK* genes in the plasma of patients with FL to evaluate the clonal heterogeneity and prognostic value of ctDNA. This technique is sensitive; a tumour clonotype could be detected in 85% of our patients in the tumour diagnostic sample. The clonotype or one of its subclones was then identified in 86% of the cases in the corresponding plasma sample. Seventeen percent of the patients (4 patients out of the 29) had no detectable tumour ctDNA, although non-tumoural *IG* rearrangements were detected in the plasma samples and a high level of calibrating clonotype (from 164,400 to 1,047,416 lymphoma clonotype molecules per million diploid genomes) was present in the tumour samples, allowing us to exclude a sensitivity-related technical issue. The immunosequencing approach allowed the detection of subclonal population(s) in the tumour and in the plasma, reflecting the clonal diversity in FL. We also showed the clinical implication of the detection of ctDNA, with patients presenting with a high ctDNA level at diagnosis having a shorter PFS than the others.

Over the past 20 years, impressive progress has been made in the treatment of FL, thanks to the introduction of anti-CD20 antibodies in combination with chemotherapy for high-tumour-burden or symptomatic patients followed by the maintenance strategy [17, 19, 20]. Nevertheless, FL remains an incurable disease, and patients still experience relapse without a clear “plateau” on the PFS curves. Different prognostic markers (FLIPI and FLIPI 2 scores [21–23]) are routinely used to predict the OS and PFS of FL patients, but these markers are not accurate enough to predict chemo-refractoriness, and treatment strategies still rely on the clinical symptomatic characteristics of the patients. Progress has been made in understanding the biology of the disease, and a new score integrating molecular parameters has been proposed (m7-FLIPI [24]). The detection and quantification of clonal ctDNA with sensitive techniques, such as NGS-based Ig sequencing (Lymphosight technology), was shown here to be a potential clinically relevant biomarker at diagnosis.

Using this NGS-based immunosequencing method, we were also able to examine the important clonal diversity in FL at diagnosis and a variation of the distribution of subclones between tumour biopsy and plasma samples. Eighteen out of the 24 patients (75%) with an *IGH-V* or *-D* clonotype had one or more detectable subclones in the tumour or in the plasma. Moreover, these subclones appear to disseminate extensively in the blood, as demonstrated by the detection of ctDNA. In 13 cases (out of 24 cases, 54%), a subclone was detected in both the plasma and the tumour. This substantial subclonal dissemination was associated with increased diversity in the number of subclones, ranging from 2 to 71 in the tumour and from 2 to 20 in the plasma. These results highlight the ongoing somatic hypermutation process previously reported in FL cells [25] and the high intra-clonal heterogeneity. The *IGK* assay allowed the detection of sub-clones in 12% (2/17 patients), and none of them disseminated in the blood. This contrasts with the results obtained with the *IGH* assays, suggesting that the detection of the light-chain clonotype may not be the most suitable marker to highlight clonal heterogeneity. We did not observe a significant impact of the clonal diversity and the numbers of detected subclones on the patients’ outcome. Nevertheless, despite the limited size of this series, patients with a high number of sub-clones tended to have a longer PFS than the others. This might be related to the longer time of lymphoma development as an occult disease, with a higher mutation rate in the lymphoma cells, potentially resulting in an enhanced immune response against tumour cells. This hypothesis needs to be further evaluated in a larger cohort with adequate exploration of the lymphoma micro-environment immune component.

The presence or absence of ctDNA in diagnostic plasma samples did not correlate with any clinical characteristics in this series except with bone marrow involvement. The absolute level of the highest frequency clone in the plasma ranged from 0 to 345.000 clonotypes per million diploid genomes and was related to the presence of circulating lymphoma cells and LDH value but not to other clinical or biological characteristics. Nevertheless, the ctDNA level of the highest-frequency clone at diagnosis had a strong prognostic value for PFS (p=.004). Furthermore, we showed that this poor prognostic value of elevated ctDNA levels in the plasma at diagnosis appeared independent of other relevant clinical parameters, such as the FLIPI score, bone marrow involvement, presence of circulating lymphoma cells or LDH value. Interestingly, rituximab maintenance could potentially abrogate the adverse prognostic value of high ctDNA at diagnosis. These results have to be confirmed and must be taken carefully given the potential biases associated with the low number of patients included in the study. Nevertheless, this is in line with our previous data regarding the adverse outcome associated with the presence of circulating lymphoma cells in FL patients, which was also erased by the addition of maintenance with rituximab [26]. This effect of rituximab maintenance on the predictive value of the lymphoma ctDNA amount underscores the notion of lymphoma cells’ persistence in lymphoma niches that may require prolonged treatment. Zohren et al [27] reported that the pre-treatment *BCL2/IGH* level in the blood assessed by PCR (*BCL2/IgH* rearrangement) also had clinical relevance. However, in this report [27], only 66% of the patients had a detectable *BCL2/IgH* rearrangement at diagnosis, while the NGS technology used in our series allowed us to obtain an assessable marker in a higher proportion of patients (85%). Consistent with our findings, these authors also describe a high inter-individual variability in *BCL2/IgH* levels among newly diagnosed FL patients and a correlation with bone marrow involvement. The prognostic value of the ctDNA level at diagnosis in our report and of the *BCL2/IGH* level in Zohren et al. are both independent from other clinical markers, such as the FLIPI score and the β2 microglobulin level. Ghielmini [28] and co-workers also found that the pre-treatment *BCL2/IGH* levels from the peripheral blood were predictive for the response duration to rituximab monotherapy.

Therefore, ctDNA detection with an NGS-based immunosequencing method may represent a more sensitive technique than qPCR-based analysis. This approach allows a description of the heterogeneity of sub-clonal populations and appears to be able to predict the outcome of FL patients at diagnosis. Nevertheless, one of the most important weaknesses of our studies is that we did not assess the post-treatment ctDNA level in the plasma from FL patients. Our data remain preliminary, but they demonstrate that the concept of ctDNA detection, as assessed with NGS immunosequencing techniques, may have a high clinical value in identifying a high-risk population of patients with FL at diagnosis.

## PATIENTS AND METHODS

The PRIMA [17] study (NCT00140582) was a prospective phase III trial involving untreated patients with high-tumour-burden FL between 2004 and 2007. Patients with grade 1, 2 or 3a FL according to the WHO classification were eligible, and tumour biopsies were centrally reviewed. In brief, patients received 6 cycles of rituximab plus cyclophosphamide, doxorubicin, vincristine and prednisone completed with 2 infusions of rituximab (R-CHOP, 885 patients); 8 cycles of rituximab plus cyclophosphamide, vincristine and prednisone (R-CVP, 272 patients); or 6 cycles of rituximab, fludarabine, cyclophosphamide and mitoxantrone (R-FCM, 45 patients). At the end of induction, the responding (complete or partial response, CR or PR) patients were randomly allocated to maintenance with rituximab, one infusion every two months for 2 years, or observation. Among the PRIMA cohort, paired tumour biopsy and plasma samples at diagnosis were available for only 34 patients who were included in the present study.

Using the NGS-based immunosequencing method (Adaptive Biotechnologies, South San Francisco, CA), the lymphoma clonotype was first established in the tumour biopsy using locus-specific primer sets for *IGH-V*, *IGH-D* and *IGK* rearrangements. In the tumour sample, clonotypes present at a frequency greater than 5% of all rearranged *IG* sequences were considered as originating from the lymphoma clone. For each assay (*IGH-V*, *IGH-D* and *IGK*), the highest frequency clone in the tumour was called the “calibrating” clonotype. The lymphoma-derived sequences identified in the biopsy sample were then used as targets to assess the presence of ctDNA in the plasma samples. To study clonal diversity, i.e., subclones or evolved clones, clones presenting point mutation(s) in the V(D)J sequence compared to the calibrating clonotype, were identified both in the tumour and in the plasma samples. A coalescence algorithm was used to distinguish the evolved or mutated clones from methodological amplification or sequencing errors. The coalescence algorithm attempts to combine related sequences that are produced by PCR or sequencing artefacts. The algorithm considers the number of reads of each sequence, the number of base differences between them and the average Q score of the differing bases. Parameters were picked so that when artificial pure templates were deeply sequenced, there were no apparent “mutated” clones. The quantity and frequency of ctDNA in the plasma was calculated relative to the total number of reads in the sample and defined as lymphoma clonotype molecules per million diploid genomes. We used *GAPDH* as a control gene to infer the total number of diploid genomes in each reaction. Quantification was performed in duplicate for two different dilutions. To assess the prognostic value of ctDNA level, the median of ctDNA level in each assay was considered as the threshold. In cases with both *IGH-V* (or *–D*) and *IGK* calibrating clonotypes and/or subclones, the median of the sum of each assay was considered as the threshold. To build hierarchical trees, the clonal frequency of each subclone in the tumour and in the plasma was assessed relative to the total number of rearranged receptors in the sample.

For statistical analysis, progression-free survival (PFS) was calculated from the time of registration in the PRIMA trial to the time of FL progression or death from any cause. The correlations between the presence of ctDNA, the ctDNA levels and the clinical or biological characteristics of the patients at diagnosis were assessed using a χ^2^ test. Statistica® software was used for statistical analysis.

The study was conducted in accordance with the local ethics committees and the declaration of Helsinki.

## Abbreviations

FLIPI: follicular lymphoma international prognostic index
LDH: lactate dehydrogenase
UNV: upper normal value
ctDNA: circulating tumour DNA in plasma samples
